# Achieving equitable leadership in Global Health partnerships: barriers experienced and strategies to improve grant funding for early- and mid-career researchers

**DOI:** 10.1186/s44263-024-00047-4

**Published:** 2024-03-08

**Authors:** Chido Dziva Chikwari, Amare Worku Tadesse, Kwame Shanaube, Anna Shepherd, Christopher Finn McQuaid, Toyin O. Togun

**Affiliations:** 1 The Health Research Unit Zimbabwe, Biomedical Research and Training Institute, Harare, Zimbabwe; 2 Department of Infectious Disease Epidemiology, Faculty of Epidemiology and Population Health, London School of Hygiene & Tropical Medicine, London, UK; 3 TB Centre, and Department of Infectious Disease Epidemiology, Faculty of Epidemiology and Population Health London School of Hygiene & Tropical Medicine, London, UK; 4 Zambart, Lusaka, Zambia; 5 TB Centre and Clinical Research Department, Faculty of Infectious and Tropical Diseases, London School of Hygiene & Tropical Medicine, London, UK; 6 Medical Research Council Unit The Gambia at the London School of Hygiene & Tropical Medicine, Atlantic Boulevard, Banjul, The Gambia

**Keywords:** Global health, Equity, Leadership, Research, Funding, Partnerships, Decolonization

## Abstract

Calls to decolonize global health have highlighted the continued existence of colonial structures in research into diseases of public health importance particularly in low- and middle-income countries (LMICs). A key step towards restructuring the system and shaping it to local needs is equitable leadership in global health partnerships. This requires ensuring that researchers in LMICs are given the opportunity to successfully secure grant funding to lead and drive their own research based on locally defined priorities. In February 2022, the London School of Hygiene and Tropical Medicine hosted a workshop aimed at bringing together funders and early- and mid-career researchers (EMCRs) to identify funder initiatives that have worked to improve equitable leadership, to better understand barriers faced by researchers, and collectively brainstorm approaches to overcome these barriers. The workshop transcript was analyzed using a deductive thematic approach based on the workshop topic to identify key emerging themes. Barriers identified were the lack of individual and institutional level support and flawed funding structures for EMCRs in LMIC settings. Strategies on how equitable leadership can be further facilitated include institutional reforms for funders to facilitate equity, diversity, and inclusion in their partners through consultative engagement and in addition, reshaping how research priorities are defined; diversified funding streams for research organizations, building partnerships and dedicated funding for capacity building of EMCRs. Intentional advances to overcome funding barriers in global health speak directly to its decolonization. Urgently required and complex changes in practice must be intentional and do require uncomfortable shifts which will take time.

**Supplementary Information:**

The online version contains supplementary material available at 10.1186/s44263-024-00047-4.

## Background

Recent calls to decolonize global health have highlighted the continued existence of colonial structures in research into diseases of public health importance in low- and middle-income countries (LMICs) in particular. While research projects are inextricably linked to informing the policy and practice for improving the health of the study population [1], grant funding for such projects also plays a critical role in defining the research priorities and leadership. As such, most of the global health research conducted in LMICs has been led by researchers from high-income countries. A key step towards restructuring the system and shaping it to local needs is the necessity for what we termed “equitable leadership in global health partnerships.” This requires ensuring that researchers in LMICs are given the opportunity to successfully secure grant funding to lead and drive their own research based on locally defined priorities. Despite initiatives from various funders, this continues to represent a major challenge in LMICs [2, 3].

In February 2022, the London School of Hygiene and Tropical Medicine (LSHTM) Tuberculosis (TB) Centre organized a Decolonising Global Health workshop, which was sub-themed “Equitable Leadership in Global Health Partnerships”. The aim of the workshop was to bring together funders and early- and mid-career researchers (EMCRs) to identify funder initiatives that have worked to improve equitable leadership, to better understand barriers faced by researchers, and to collectively brainstorm approaches to overcome these barriers. The virtual workshop was attended by more than 140 people (127 completed the workshop survey) representing funders, government agencies, and EMCRs from Africa, Europe, Asia, and South America (Table 1). The researchers represented diverse areas of interest including TB, Malaria, HIV, COVID-19, antimicrobial resistance, non-communicable diseases, and neglected tropical diseases. Speakers at the workshop included senior representatives from higher education and research institutions, EMCRs from LMIC or doing their research in LMICs, representatives from funding agencies, and other stakeholder organizations. This manuscript adds to the existing literature on research capacity strengthening and global inequalities by providing perspectives from both researchers and funders through dialogue as well as agreed-up recommendations for change [4, 5].

**Table 1:** Workshop participants’ characteristics

Variable		***N* = 127** ***n* (%)**
Gender	Male	74 (58)
	Female	51 (40)
	Other/do not want to say	2 (2)
Location	Africa	73 (58)
	Asia	7 (6)
	Europe	43 (34)
	Other	4 (3)
Research area^a^	TB	42 (33)
	COVID-19	17 (13)
	HIV	26 (21)
	Non-communicable diseases	14 (11)
	Antimicrobial resistance	17 (13)
	Sexual and reproductive health	14 (11)
	Malaria	20 (16)
	Other	39 (31)
	Not a researcher	15 (12)
Career level (if researcher)	Pre-PhD	22 (17)
	PhD student	22 (17)
	Early post-doc (< 4 years post-PhD)	17 (13)
	Senior post-doc (> 4 years post-PhD)	22 (17)
	Senior Scientist	19 (15)
	Not applicable	25 (20)

^a^Multiple research areas selected by some participants—overall tally greater than the total number of participants

The workshop had two sessions; a session on the researcher’s experiences with barriers, and a session on approaches to overcome the barriers (Additional file 1: Workshop Programme). Each session included presentations from both funders and researchers, as well as consultative sessions which were open to the audience to share views and experiences, discuss, and ask questions of the presenters. The workshop was recorded, and the workshop transcript was analyzed using a deductive thematic approach based on the workshop topic to identify key emerging themes.

We hereby summarize the key themes that emerged from the workshop on barriers and challenges in securing grant funding experienced by EMCRs from LMICs, and potential strategies to mitigate these barriers, as well as provide recommendations to address inequities in funding for global health research. We also provide supporting quotes from the participants to buttress the points made.

## Barriers experienced by early- and mid-career researchers

**Fig. 1:**
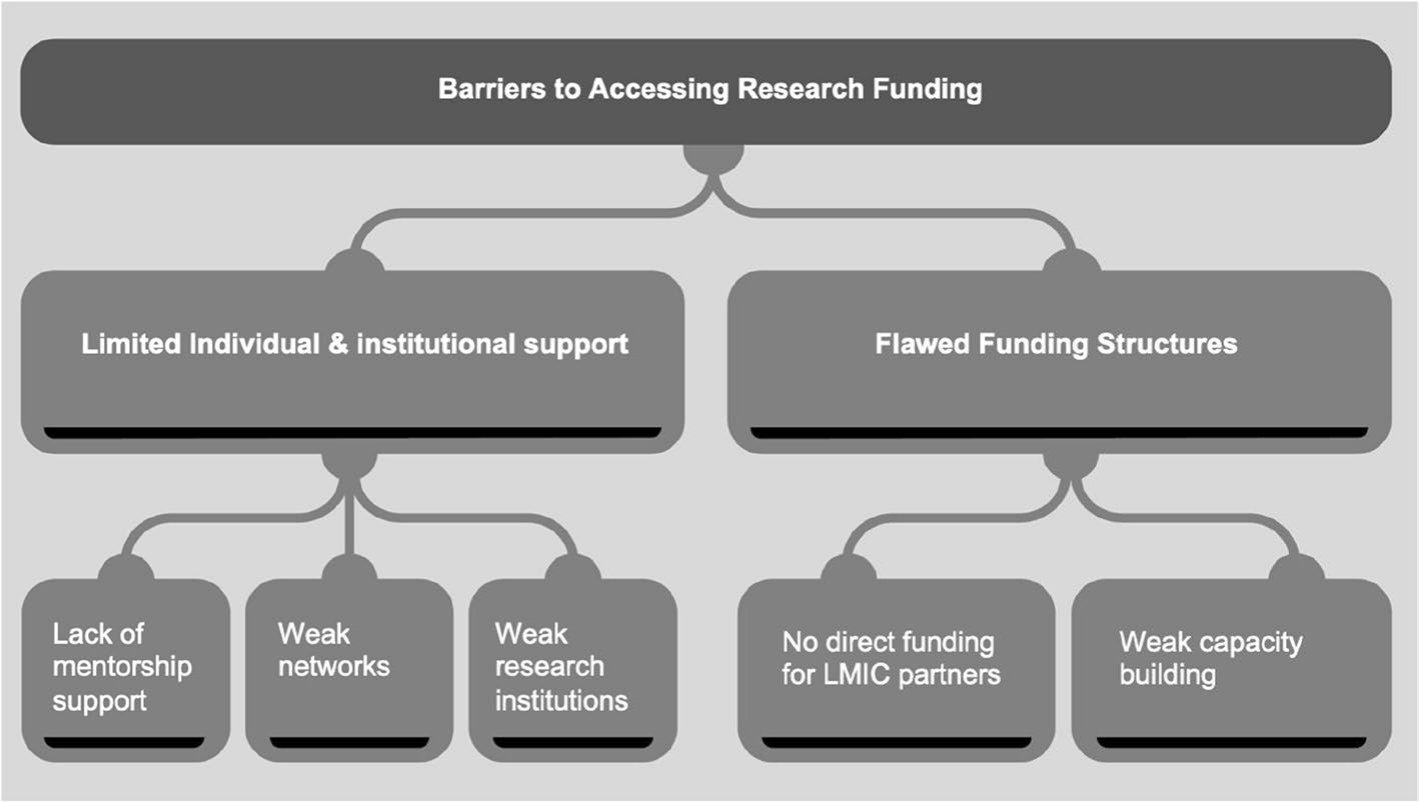
Barriers to accessing research funding experienced by early- and mid-career researchers

To address equity in research funding it is critical that barriers to accessing funding be addressed. Key barriers to accessing research funding for EMCRs from LMICs identified were grouped into two main themes: specifically, the lack of individual and academic/research institutional level support, and flawed funding structures for EMCRs in these settings (Fig. 1).

## Lack of individual and institutional-level support

### Lack of mentorship support

Mentorship plays a critical role in guiding EMCRs in the development of their research career path, and also in the formulation of research ideas and future leadership [6]. EMCRs, who are often at the start of transitioning to independence from direct supervisors, identified a lack of mentorship support in how to maintain and build on that independence as a key gap that if addressed could support these EMCRs and strengthen their networks.

“As you are beginning you need to work with people that can actually help build you.”

EMCR, Female

### Weak networks

In addition to support through mentorship, EMCRs identified the need for strong networks to assist them in the grant writing and funding application process.

“One of the key pillars of my success has been the ability to be able to link into international networks.”

EMCR, Male

These networks, teams, and institutional pathways for progression facilitate the generation of ideas with researchers who are already established and recognized. They also allow for the building of multidisciplinary teams of collaborators with cross-pollination of skills, experience, and ideas which would facilitate improving both their ability to conduct research and also the confidence of funders.

“Linking with networks is important because current research questions can’t be answered by a single institution. There needs to be a consortium whether it’s an institution or individuals that cut across different sectors.”

EMCR, Male

### Weak research institutions

It was also highlighted that, in addition to individual-level support, institutional support and infrastructure are often a potential barrier in many research institutions. For example, some research institutions were identified as having strong structures, support, and pathways for EMCRs to secure research funding, with associated improved success rates.

“Certain institutions have better applications, so it looks as if the funding is targeted for them. So (for other institutions) there must be some institutional commitment towards grant applications.”

EMCR, Female

Senior researchers noted that training in grantsmanship is not available in all research institutions, and this institutional shortcoming could have a direct impact on the quality and competitiveness of grant applications particularly for researchers in the postdoctoral stage.

“Turning a good research idea into a fundable research project remains a problem.”

Funding agency representative, Male

## Flawed funding structures

### No direct funding for LMIC partners

In addition to the requirement for individual and facility-level support for EMCRs, another key barrier identified was flaws in existing research funding. Critical among these flaws was the fact that there are few available funding sources for EMCRs, with additional gender biases against women [7].

“Funding opportunities for early career researchers are few and far between, and the bias more pronounced in LMICs.”

EMCR, Male

Large funders were reported to not fund LMIC partners directly.

“We know that not all research funders are willing to fund LMIC researchers directly.”

Research Institution representative, Female

Where research partnerships were proposed as a solution to address barriers in direct funding to LMIC, these partnerships were frequently not equitable.

### Weak capacity building

Investments in health programs in LMICs were reported to largely benefit health but not necessarily capacity for knowledge generation. Barriers to funding for researchers in the early post-doctoral period were identified, as part of the challenge is getting trained researchers to remain within LMICs. In addition, when funding opportunities have been unsuccessful, EMCRs also highlighted the need for funders to provide constructive feedback which is not often made available but plays a critical role in strengthening future applications.

“The feedback that you get from most of these donors is not really constructive enough to help improve future applications.”

EMCR, Male

## Strategies to address barriers

**Fig. 2:**
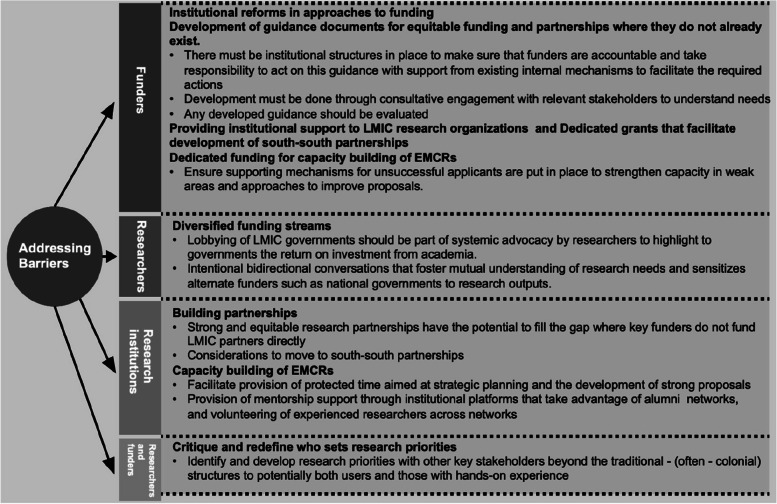
Strategies to address barriers

Both representatives of funders and researchers who participated in the workshop identified several strategies on how equitable leadership can be further facilitated through research funding for EMCRs from LMICs. Critical strategies to address the barriers in funding and thus equitable leadership in Global Health partnerships included academic/research institutional reforms for funders to facilitate equity, diversity, and inclusion in their partners through consultative engagement and in addition, reshaping how research priorities are defined, diversified funding streams for research organizations, building partnerships and dedicated funding for capacity building of EMCRs (Fig. 2).

## Institutional reforms

The role of funders in shifting and influencing research partnerships is central to the development of equitable leadership. This could begin with funder-level institutional reforms in the approaches to funding that are to be taken by funders and the development of guidance documents for equitable funding and partnerships where they do not already exist within funding bodies. In the rare instances guidance documents developed by funders do exist, it was noted that there must be funder-level institutional structures in place to make sure that funders themselves are accountable and take responsibility to act on this guidance with support from existing internal mechanisms to facilitate the required actions.

Although it is now very attractive for both funding agencies and research institutions to have ‘Equity, Diversity, and Inclusion (EDI) action plans’, gaps in their implementation, monitoring, and evaluation were noted. These EDI action plans need to be acted on together with ongoing reviews of the policies and procedures to support them as well as evaluations of their effectiveness.

### Consultative engagement

Where funder institutional mechanisms to support equitable leadership have not been put in place, funders were urged to seek to understand the needs through consultative processes that will engage all relevant stakeholders. Any interventions or programs that have been developed and implemented should also be evaluated to ensure they are producing the intended outcomes and have an impact.

A funding agency that had recently completed its consultative process shared findings and a template of key elements that funders should consider in their institutional guidance, focusing not only on the hardware (policies and frameworks) of research funding but also the software (power, respect, due diligence, context) [8].

## Defining research priorities

As part of funder institutional reforms, funders were also challenged to critique and redefine who defines research priorities. Research priorities are mostly not developed by those who use the research. As such, funders and researchers were encouraged to identify and develop research priorities with other key stakeholders beyond the traditional (often colonial) structures to potentially both users and those with hands-on experiences [9]. As part of the software in research funding, the power dynamics of deciding research priorities should be evaluated.

“In our positions, we are gatekeepers, we are also sort of perpetuating the same power dynamics with our stakeholders and our partners.” Senior Researcher, Female

## Diversified funding streams

An additional theme with prominence in the workshop was the importance of researchers exploring funding from other avenues, including government funding. This includes intentional bidirectional conversations that foster a mutual understanding of research needs and sensitize these alternate funders to research outputs. Engaging LMIC governments should be part of systemic advocacy by researchers to highlight to governments the return on investment from academia.

“Our research questions need to be aligned to national priorities. […] We need to align our research ideas to exactly what the government wants.”

EMCR, Female

### Building partnerships

Strong and equitable research partnerships have the potential to fill the gap where key funders do not fund LMIC partners directly. It is important to note, however, that at present these research partnerships are dominantly north–south partnerships, which are often not equitable [10].

Similarly, decolonizing research in LMICs requires major funders to create schemes that explicitly request and support south-south collaboration. Current funding structures, and grant calls which often require a “northern collaborator” tend to give the impression that the northern institutions (both collaborators and funders) are driving the research as is often the case. As such, providing institutional support for LMIC research organizations was also identified as a potential strategy to mitigate the funding barriers for research.

“A careful look at what we've done to date shows that we focus ourselves on individuals. Now having developed the right kind of individuals we should also focus on institutions.”

Funding Institution Representative, Male

LMIC organizations often do not have funding to facilitate partnerships, specifically south-south partnerships. The current model of north–south partnerships is often linked to specific projects and does not respond directly to the needs of local research institutions. It tends not to be geared towards building capacity and retaining skilled researchers locally. Dedicated grants that facilitate the development of south-south partnerships could also allow smaller organizations to collaborate and bid for larger grants together with more established intuitions.

“We have to value the partnership from the South, we have to respect the autonomy, we have to ensure that they have agency.”

EMCR, Male

### Dedicated funding for capacity building of EMCRs

In addition to guidance for EDI, funder initiatives should also ensure distributed funder arrangements which include ringfenced funding for capacity building for EMCRs. Such capacity-building funds could support researcher development fellowships and platforms for networking, such as alumni networks and resource hubs set up by funders to allow for synergy. Funders present in the workshops shared examples of existing programs such as preparatory fellowships and development programs consisting mainly of essential skills training for grant writing and peer review workshops.

“We seek early outcomes to ensure that these fellows have developed new skills and new networks.”

Funding Institution Representative, Male

Within funding schemes, funders were also urged to ensure that support mechanisms for unsuccessful applicants are put in place to strengthen capacity in weak areas and approaches to improve proposals*.*

In addition to wider capacity-building initiatives to address some of the support needs of EMCRs, it was highlighted that research institutions should facilitate the provision of dedicated time to allow for grant writing because EMCRs are often also busy with teaching, direct project management, and fieldwork. This protected time would then be aimed at strategic planning, which is pivotal to the development of strong proposals.

Among EMCRs themselves, an improved skillset would improve their eligibility and facilitate flexibility for geographical and institutional mobility, allowing relocation to institutes with better networks and opportunities for career development. The intentional provision of mentorship support through research/academic institutional platforms that take advantage of alumni networks, and volunteering of experienced researchers across networks, was also flagged as a possible strategy to fill the mentorship gap and thereby build capacity.

## Conclusions

Despite existing best practice initiatives already being implemented in LMICs, EMCRs continue to face barriers to accessing research funding which is the cornerstone for equitable leadership in global health [11–13]. This workshop report highlights some key prevailing barriers for EMCRs from LMICs, such as a lack of mentorship support, weak networks, and poor research/academic institutional support. Ongoing and successful strategies to build capacity, and provide mentorship and networks are commended [14–16]. These initiatives although few and far apart are paramount to learn from and build upon in order to strengthen them and develop sustainable impact. Evaluations of their effectiveness should also be prioritized.

The role of funders in continuing to address these barriers is critical. Although some funder initiatives have been established and reviews published, these could be improved by facilitating the sharing of resources and power specifically through redefining who sets out research priorities, ringfenced funding for EMCRs from LMICs, and providing institutional support at all levels to enable collaborative south-south partnerships and capacity building [12, 13, 17]. Additional funding avenues for research in LMICs should be explored such as local government funding that meets commitments made in the 2001 Abuja meeting and LMIC-based philanthropic funding which at present does not play a major role in the funding landscape [18, 19]. In addition, research institutions and researchers themselves have a role to play and would benefit from widened engagement with stakeholders, including local governments, and fostering collaborations with other local and international organizations, to strategically place themselves to obtain research funding and to address locally relevant research priorities.

A key limitation of our paper is that we did not differentiate between challenges faced by researchers based in LMICs and those working in LMICs but based in non-LMIC settings. Challenges experienced by these two groups are likely overarching although separation of these may have provided additional nuance. An additional limitation to note is that this manuscript did not aim to provide a resource or review of currently existing best practices by name but merely provided perspectives from represented groups. Despite this such resources do exist such as those provided by many funding programs in recent years.

**Fig. 3:**
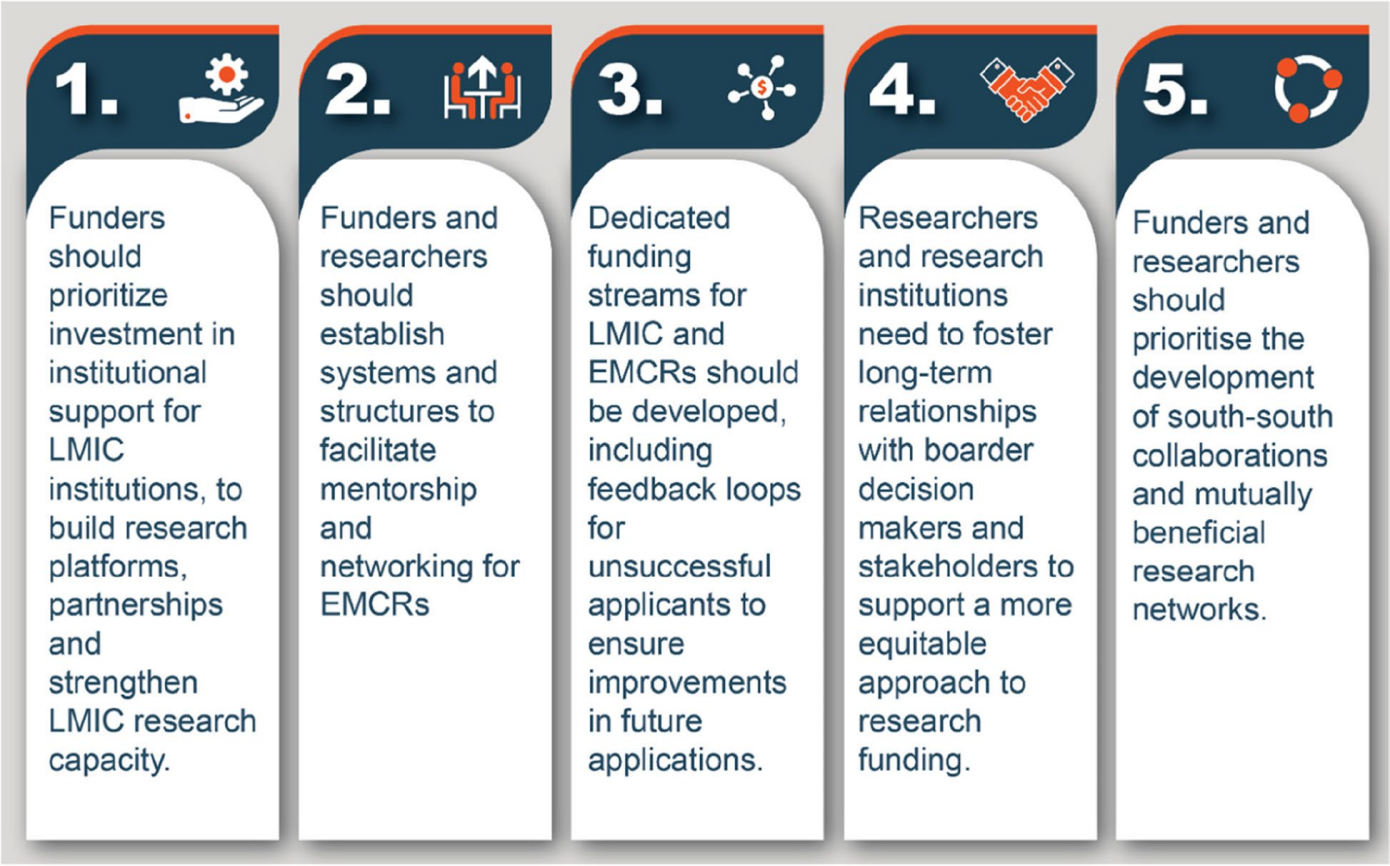
Recommendations to improve access to research funding for early- and mid-career researchers

Overall, intentional advances to overcome funding barriers in global health speak directly to its decolonization. However, it is important to note that these changes despite their complexity must be intentional and do require uncomfortable shifts in thinking which will take time from funders and researchers. We provide 5 key and urgent recommendations for reform for funders, researchers, research organizations, EMCRs, and other stakeholders to achieve equitable research partnerships (Fig. 3):

1. Funders should prioritize investment in institutional support for LMIC research/academic institutions, to build research platforms and partnerships and strengthen LMIC research capacity.
2. Funders and researchers should establish systems and structures to facilitate mentorship and networking for EMCRs
3. Dedicated funding streams for LMIC and EMCRs should be developed, including feedback loops for unsuccessful applicants to ensure improvements in future applications.
4. Researchers and research institutions need to foster long-term relationships with boarder decision-makers and stakeholders to support a more equitable approach to research funding.
5. Funders and researchers should prioritize the development of south-south collaborations and mutually beneficial research networks.

With institutional actions geared towards delivering on these recommendations, in future years we can hope to achieve equitable leadership in global health, directly facilitated by those who are at present EMCRs.

## Supplementary Information

**Additional file 1.** Workshop Programme.

## Data Availability

A recording of the session is available online: https://www.lshtm.ac.uk/newsevents/events/equitable-leadership-global-health-partnerships

## Abbreviations

EDI: Equity, Diversity, and Inclusion
EMCRs: Early- and mid-career researchers
LMICS: Low- and middle-income countries
LSHTM: London School of Hygiene and Tropical Medicine
