# Publisher Correction: Achieving equitable leadership in Global Health partnerships: barriers experienced and strategies to improve grant funding for early- and mid-career researchers

**DOI:** 10.1186/s44263-024-00064-3

**Published:** 2024-05-08

**Authors:** Chido Dziva Chikwari, Amare Worku Tadesse, Kwame Shanaube, Anna Shepherd, Christopher Finn McQuaid, Toyin O. Togun

**Affiliations:** 1 The Health Research Unit Zimbabwe, Biomedical Research and Training Institute, Harare, Zimbabwe; 2 Department of Infectious Disease Epidemiology, Faculty of Epidemiology and Population Health, London School of Hygiene & Tropical Medicine, London, UK; 3 TB Centre, and Department of Infectious Disease Epidemiology, Faculty of Epidemiology and Population Health London School of Hygiene & Tropical Medicine, London, UK; 4 Zambart, Lusaka, Zambia; 5 TB Centre and Clinical Research Department, Faculty of Infectious and Tropical Diseases, London School of Hygiene & Tropical Medicine, London, UK; 6 Medical Research Council Unit The Gambia at the London School of Hygiene & Tropical Medicine, Atlantic Boulevard, Banjul, The Gambia

**Publisher Correction: BMC Global Public Health 2, 17 (2024)**

**https://doi.org/10.1186/s44263-024-00047-4**

Following publication of the original article [1], a typesetting error was noticed in the competing interests declaration, whereby the caption of the supplementary file was mistakenly typeset.

Incorrect competing interests: Additional file 1. Workshop Programme.

Correct competing interests: The authors declare no competing interests.

The original article [1] has been corrected.
