# Supplementary material for: Achieving equitable leadership in Global Health partnerships: barriers experienced and strategies to improve grant funding for early- and mid-career researchers
Source: BMC Glob Public Health. 2024 Mar 8;2:17. doi: 10.1186/s44263-024-00047-4 (PMC11078704; doi:10.1186/s44263-024-00047-4)
Supplement: Supplementary file 1 — Additional file 1. Workshop Programme. [file 44263_2024_47_MOESM1_ESM.docx]

**LSHTM TB Centre Decolonising Global Health Workshop**

**Theme: *“Equitable leadership in global health partnerships.”***

Date: **22 February 2022**

Format: **Two-part webinar**

Time: **11:00 – 14:45 GMT**

[11.00-12.25: Session 1 - Researchers' experience of remaining barriers](https://www.lshtm.ac.uk/newsevents/events/equitable-leadership-global-health-partnerships)

- 11.00-11.05: Opening remarks
  - Speaker: [Dr Toyin Togun](https://www.lshtm.ac.uk/aboutus/people/togun.toyin), Co-Director of the LSHTM TB Centre and Associate Professor, LSHTM
- 11.05-11.10: Welcome address
  - Speaker: [Professor Anne Mills](https://www.lshtm.ac.uk/aboutus/people/mills.anne), Deputy Director & Provost, LSHTM and Professor of Health Economics and Policy, LSHTM
- 11.10-11.25: Presentation 1 - Overview of DGH of research as an issue
  - Speaker: [Dr Catherine Kyobutungi](https://aphrc.org/person/catherine-kyobutungi/), Executive Director, African Population & Health Research Centre
- 11.25-11.40: Presentation 2 - Sharing of current and planned funder initiatives to address DGH issues
  - Speakers:  [Amen-Patrick Nwosu](https://www.linkedin.com/in/amennwosu?originalSubdomain=uk) and [Alice Chadwick El-Ali](https://uk.linkedin.com/in/alicechadwick), The UK Collaborative on Development Research (UKCDR)
- 11.40-12.25: Consultative session - Early/mid-career researchers (EMCRs) to discuss and share experience of barriers to securing grant funding to lead and drive their own research based on locally defined priorities.
  - Moderators:
    - [Dr Chido Dziva Chikwari](https://www.lshtm.ac.uk/aboutus/people/dziva-chikwari.chido), Assistant Professor of Epidemiology, LSHTM
    - [Dr Toyin Togun](https://www.lshtm.ac.uk/aboutus/people/togun.toyin), Co-Director of the LSHTM TB Centre and Associate Professor, LSHTM

[13.00-14.45: Session 2 - Approaches to overcome these barriers](https://www.lshtm.ac.uk/newsevents/events/equitable-leadership-global-health-partnerships)

- 13.00-13.30: Presentation 3 - Selected researchers experience of what works
  - Speakers:
    - [Professor Moses Bockarie](http://www.edctp.org/about-us/edctp-secretariat/professor-moses-john-bockarie/), Director of International Cooperation Africa & Head of Africa Office of European and Developing Countries Clinical Trials Partnership
    - [Professor Tumani Corrah](https://www.africaresearchexcellencefund.org.uk/about-us), Co-President, Africa Research Excellence Fund
- 13.30-14.15: Consultative session and Q&A - Identification of approaches to overcome the identified barriers from the perspectives of EMCRs.
  - Moderators:
    - [Professor Rashida Ferrand](https://www.lshtm.ac.uk/aboutus/people/ferrand.rashida), Professor of International Health, LSHTM
    - [Dr Finn McQuaid](https://www.lshtm.ac.uk/aboutus/people/mcquaid.finn), Co-Director of LSHTM TB Centre and Assistant Professor in Infectious Disease Epidemiology, LSHTM
- 14.15-14.40: Plenary discussion - Synthesis of suggestions and identification of next steps
  - Moderator: [Dr Chido Dziva Chikwari](https://www.lshtm.ac.uk/aboutus/people/dziva-chikwari.chido), Assistant Professor of Epidemiology, LSHTM
  - Panellists:
    - [Dr Amare Tadese](https://www.lshtm.ac.uk/aboutus/people/tadesse.amare), Assistant Professor, LSHTM
    - [Dr Kwame Shanaube](http://www.zambart.org.zm/leadership/zambart-team/), Deputy Director of Research, ZAMBART
    - [Dr Mishal Khan](https://www.lshtm.ac.uk/aboutus/people/khan.mishal), Associate Professor of Health Policy & Systems Research, LSHTM
- 14.40-14.45: Closing
  - Speaker: [Dr Finn McQuaid](https://www.lshtm.ac.uk/aboutus/people/mcquaid.finn), Co-Director of LSHTM TB Centre and Assistant Professor in Infectious Disease Epidemiology
